# Native pulmonary valve massive endocarditis in a drug-addicted patients with Covid-19 pneumonia: a case report

**DOI:** 10.1186/s43044-023-00421-x

**Published:** 2023-11-30

**Authors:** Andrea Garatti, Andrea Daprati, Lorenzo Lora Ronco, Carlo Gaetano Sassi, Carlo De Vincentiis

**Affiliations:** Cardiac Surgery Unit, Cardiac Surgery Division, Department of Cardiovascular Disease “E. Malan”, IRCCS Policlinico S. Donato, Via Morandi 30, S. Donato Milanese, 20097 Milan, Italy

**Keywords:** Endocarditis, Pulmonary valve, Covid-19 pneumonia, Case report

## Abstract

**Background:**

Pulmonary valve (PV) infective endocarditis is a rare pathology. Association between acute endocarditis and Covid pneumonia is equally poorly investigated.

**Case presentation:**

We present the case of a 50-year-old male active drug-abuser admitted for native PV endocarditis with huge and mobile vegetations and a concomitant interstitial SARS-Cov2 pneumonia. Surgical timing was carefully evaluated, and the patient was first treated with Remdesivir to prevent ARDS evolution. After 5 days he underwent PV replacement with bioprosthesis via patch enlargement of RVOT and a tricuspid valve De-Vega annuloplasty. The postoperative course was uneventful with complete resolution of sepsis and viremia.

**Conclusions:**

The association between infective endocarditis and Covid pneumonia is emerging in the recent months. The reorganization in cardiac surgery hub centers resulted in an increase of urgencies referral, with consequent relative observation of some pathologies (i.e., endocarditis). The widespread administration of antibiotics and corticosteroids during the first phase of the pandemic could have contributed to the development of a moderate immunodepression of the general population and, during the pandemic, patients have been reluctant to access to hospital care, and this diagnostic delay could contribute to misdiagnosis or late presentation. We believe that in the present case, the strategy of immediate viral and respiratory stabilization, followed by a timely surgical procedure, allowed an excellent outcome in a very complicated situation.

## Background

Pulmonary valve (PV) infective endocarditis (IE) is a rare entity, accounting for 1.5–2% of IE cases [1]. Published literature describing characteristics of patients with PV IE is limited to a few case series and case reports, some published in the early 90’s [2]. Right-sided infective endocarditis is strongly associated with intravenous drug abuse, although central venous catheters, alcoholism, and congenital heart disease are also major risk factors [3]. Right-sided IE management is less codified compared to the same etiology involving the left heart valves, also due to the relative lower risk of catastrophic embolic complications. In large series, only 5% to 16% of right-sided IE eventually require surgical intervention, which is nevertheless required in case of large vegetations with impending risk of massive pulmonary embolism or right heart failure [4]. Association between acute endocarditis and Covid pneumonia is equally poorly investigated [5]. It should be emphasized that Covid-related symptoms (fever, asthenia, and dyspnea) could mask underlying bacterial infection, while diagnostic tools (i.e., transesophageal echo) could be underused for safety issue and Covid-related therapy (i.e., corticosteroids and/or immunosuppressors) can exacerbate sepsis evolution. As surgical risk is never negligible in septic patients, surgical decision making in the contest of a patient simultaneously affected by IE and Covid pneumonia can become particularly troublesome. Indeed, several reports of emergent cardiac surgery performed in patients affected by Covid pneumonia reported high intraoperative mortality and perioperative respiratory complications [6]. We present the case of a 50-year-old male active drug-abuser admitted for native PV endocarditis and a concomitant interstitial SARS-Cov2 pneumonia.

## Case presentation

**Fig. 1:**
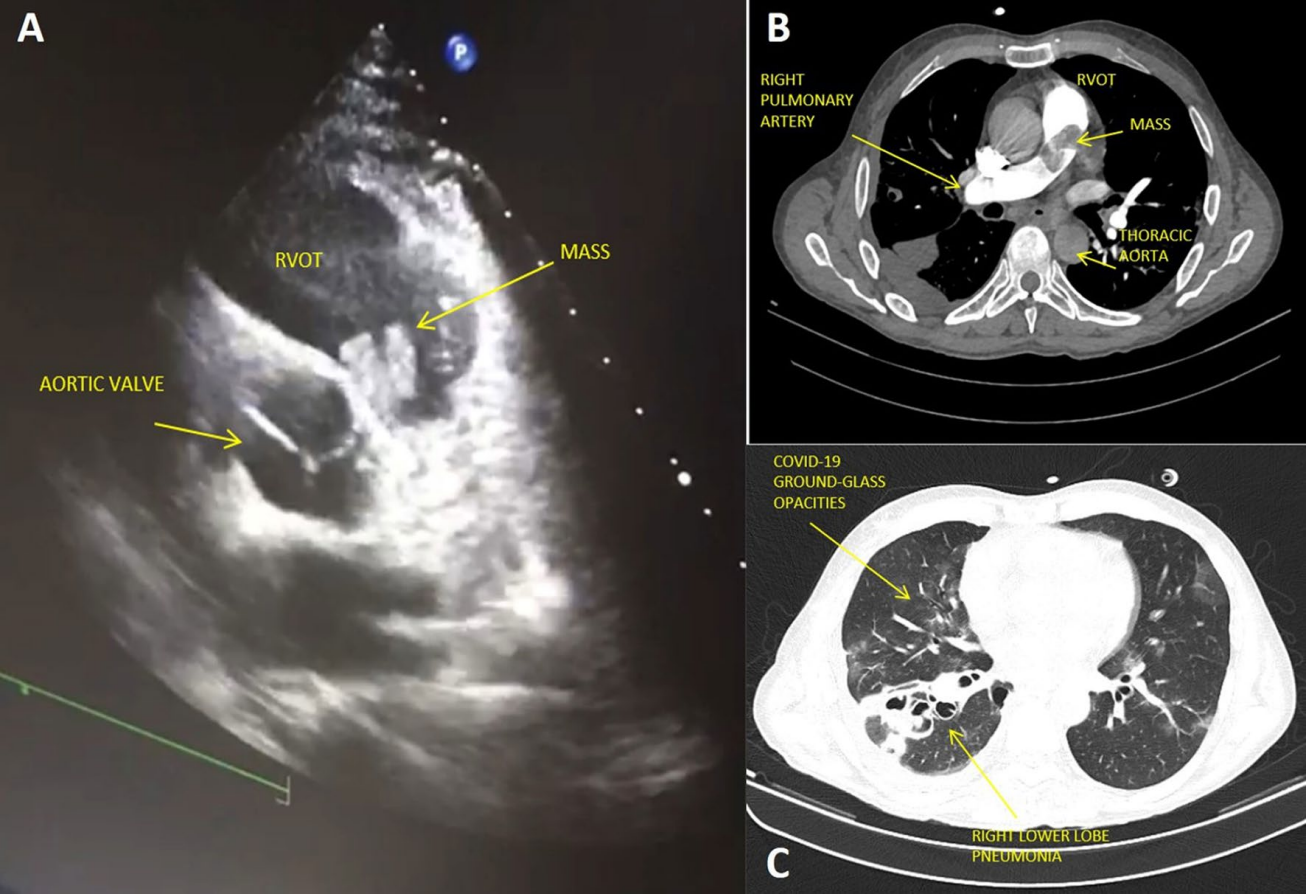
**A** Transthoracic echocardiogram showing PV mass protruding in the RVOT. **B** Chest CT scan showing PV mass extending from the RVOT to the right pulmonary artery. **C** Chest CT scan showing interstitial pneumonia with ground-glass opacities and right lower pneumonia with confluent abscesses

**Fig. 2:**
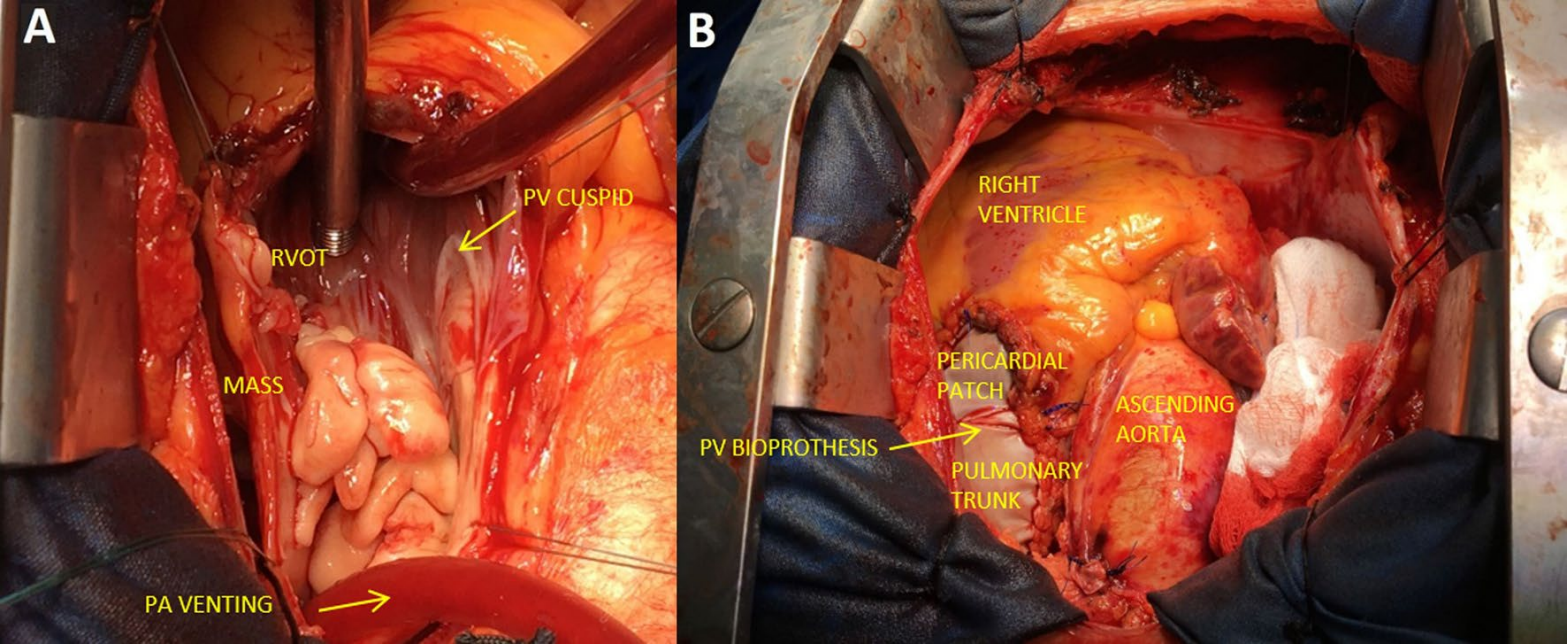
**A** Intraoperative view, longitudinal incision of pulmonary trunk showing huge endocarditic mass on PV leaflets. **B** Intraoperative view, implanted pulmonary bioprosthesis with enlargement pericardial patch on RVOT and pulmonary trunk

A 50-year-old male active drug-abuser was admitted for persistent fever, asthenia and moderate dyspnea lasting for 10 days. The cardiac tones were arrhythmic, with evidence of atrial fibrillation at the EKG, and the lungs' vesicular murmur was reduced with diffuse noises at chest auscultation, absence of skin clinical manifestations and treatable abdomen. The SARS-Cov-2 swab tested positive, and three blood cultures, obtained from different peripheral veins, resulted positive for Staphylococcus Aureus. Transthoracic echocardiography detected a huge mobile mass (24 × 17 mm) adherent to the pulmonary valve, protruding through the RVOT in the pulmonary trunk (Fig. 1A, B). The tricuspid valve (TV) presented with severe regurgitation without evidence of vegetations. Chest CT scan showed bilateral interstitial pneumonia with ground-glass opacities, and superimposed right lower lobe pneumonia, probably related to septic embolization (Fig. 1C). Despite the risk of massive pulmonary embolization, we deferred surgery in order to stabilize the viral pictures and to avoid a possible ARDS evolution, which could be exacerbated by immediate surgery [sternotomy and extracorporeal circulation(ECC)]. The patient was treated with low-dose cortisone (methylprednisolone 40 mg/die I.V.), low-molecular weight Heparin (4000 I.U. twice/day), and Remdesivir (100 mg/die) for 5 days. Respiratory support was achieved with Venturi Mask [fractional inspired oxygen (FiO_2_) = 60%] without signs of impending respiratory failure [partial oxygen pressure (pO_2_) = 115 mmHg; partial pressure of carbon dioxide (pCO_2_) = 35 mmHg; P/F ratio = 219] nor signs of ARDS evolution at a control CT scan. Surgery was then planned via median sternotomy, and ECC was instituted by means of ascending aorta and bicaval cannulation. Without cardiac arrest, pulmonary trunk was opened and a huge vegetation attached to the posterior PV leaflet was removed (Fig. 2D). A 25 Edwards Perimount bioprosthesis (Edwards Lifesciences, Irvine, CA) was then implanted, enlarging the RVOT with a heterologous pericardial patch. Finally, a TV De-Vega annuloplasty was performed without prosthetic ring, in order to limit the use of foreign material, considering the habit of intravenous drug addiction (Fig. 2E). The postoperative course was uneventful, and he was discharged from ICU in second POD. Culture-driven antibiotic therapy (Linezolid 600 mg twice/day and Oxacillin 12 g/die I.V.) was continued for six weeks with progressive clinical and infective stabilization. The patient was discharged on 28th day after surgery, from the cardiopulmonary rehabilitation center with normal cardiopulmonary objectivity, negative blood cultures and improved chest CT.

## Discussion

In our opinion there are some issues in the present experience that deserve consideration: (1) although active drug addiction is a strong risk factor, native PV endocarditis, especially without TV involvement, is an extremely rare entity (incidence of nearly 1%). (2) The association between active endocarditis and Covid pneumonia is emerging, especially in tertiary hospitals like our center, which during pandemic acted as cardiac surgery hub for the Milan metropolitan area. (3) Covid disease can partially mask subtle endocarditis signs and symptoms, considering that some diagnostic tools are underused for safety reasons and that Covid-19 therapies, like corticosteroids, can worsen septic course of the disease. 4) On the opposite, surgical timing can be difficult to establish in Covid-19 patients, as full sternotomy, coagulation dysregulation and extracorporeal circulation-related inflammatory response can exacerbate ARDS evolution of Covid-19 pneumonia.

The association between infective endocarditis and Covid pneumonia is emerging in the recent months [7]. Although a direct linkage between viral and bacterial etiology cannot be clearly established, some considerations can be ruled out: (1) the reorganization in cardiac surgery hub centers resulted in an increase of urgencies referral, with consequent relative observation of some pathologies (i.e., endocarditis, aortic dissection). (2) The widespread administration of antibiotics and corticosteroids during the first phase of the pandemic could have contributed to the development of a moderate immunodepression of the general population. (3) During the pandemic, patients have been reluctant to access to hospital care, and this diagnostic delay could contribute to misdiagnosis or late presentation.

Patients with active Covid infection and various degrees of respiratory impairment who are referred for urgent cardiac surgery pose unique challenges, especially in determining the correct surgical timing.

Indeed, several reports of emergent cardiac surgery performed in patients affected by Covid pneumonia reported high intraoperative mortality and perioperative respiratory complications. On the contrary, the delay in surgical treatment of patients simultaneously affected by IE and Covid pneumonia can result in catastrophic consequences.

## Conclusions

We believe that in the present case, the strategy of immediate viral and respiratory stabilization, followed by a timely surgical procedure, allowed an excellent outcome in a very complicated situation. As Covid pandemic is still present in several parts of the world (depending on the availability of vaccines, on the rate of no-vax, on the different government strategies for population protection), the number of Covid patients presenting to the hospitals for elective or urgent surgical procedures is going to increase. Further studies are therefore needed to optimize the surgical timing, the respiratory therapy and the perioperative management in order to improve the outcomes in this subgroup of patients.

## Data Availability

All data are available and can be shared.

## Abbreviations

PV: Pulmonary valve
ARDS: Acute respiratory distress syndrome
RVOT: Right ventricle outflow tract
IE: Infective endocarditis
TV: Tricuspid valve
ICU: Intensive care unit
POD: Postoperative day
